# Microscopic anthropogenic waste ingestion by small terrestrial European passerines: evidence from finch and tit families

**DOI:** 10.1038/s41598-025-01608-9

**Published:** 2025-05-13

**Authors:** Krzysztof Deoniziak, Anna Winiewicz, Marta Nartowicz, Weronika Mierzejewska, Sławomir Niedźwiecki, Wojciech Pol, Alina T. Dubis

**Affiliations:** 1 Division of Biodiversity and Behavioural Ecology, Faculty of Biology, University of Bialystok, Konstantego Ciołkowskiego 1J, 15-245 Białystok, Poland; 2 The Włodzimierz Chętnicki Biological Science Club, Faculty of Biology, University of Bialystok, Konstantego Ciołkowskiego 1J, 15-245 Białystok, Poland; 3 Glass Traps Foundation, Brzezina 10, 55-330 Brzezina, Poland; 4 Department of Water Ecology, Faculty of Biology, University of Bialystok, Ciołkowskiego 1J, 15-245 Białystok, Poland; 5 Faculty of Chemistry, University of Bialystok, Konstantego Ciołkowskiego 1K, 15-245 Białystok, Poland

**Keywords:** Microfibers, Microplastics, Cellulosic fibers, Terrestrial ecosystems, Raman microspectroscopy, Birds, Ecology, Zoology, Environmental sciences

## Abstract

Microscopic anthropogenic waste (MAW) has become a major environmental concern worldwide. Our study aimed to assess the accumulation of MAW in the gastrointestinal tracts of nine common European passerine species from finch (Fringillidae) and tit (Paridae) families, and evaluate their suitability for environmental monitoring. We searched for MAW in the birds’ stomachs and intestines and identified suspected particles using Raman microspectroscopy. In total, we found 57 MAW particles in 31 out of 149 analyzed individuals, 7 of which were microplastics (polyethylene, polyethylene terephthalate, polystyrene), 1 was identified as carbon nanotube, while 49 were cellulosic-based (cotton, cellulose, rayon, viscose). The generalized linear mixed models identified bird family and time in season as significant predictors of MAW ingestion. Finches ingested more MAW than tits, and higher ingestion rates were observed during the non-breeding period. Other predictors, including bird sex, age, gastrointestinal tract section, and site, showed varying but non-significant effects. As predicted, the studied species exhibited a lower ingestion rate of MAW compared to terrestrial birds studied so far, possibly due to their diet and feeding behavior. Given that these species are prey for many avian and non-avian predators, they may contribute to the transfer of MAW to higher trophic levels.

**Supplementary Information:**

The online version contains supplementary material available at 10.1038/s41598-025-01608-9.

## Introduction

Microscopic anthropogenic waste (MAW) pollution refers to the presence of small plastic particles, such as microplastics (MPs)^1,2^, and artificial cellulosic microfibers (AFs)^3,4^, present in the environment due to human activity. MPs (< 5 mm in length) originate from originally designed fragments used in cleaning and personal care products, among others, from engineered plastic pellets, and from the breakdown of larger plastic debris which undergoes weathering^5^. AFs (with a diameter usually less than 10 μm), reported as the most common type of MAW in the environment, are largely generated by the textile and garment processing industry chain, but also by domestic washing machines and dryers of clothes^3,6,7^. While total environmental pollution from MAW cannot be accurately determined, estimates show that annually over 300 tons of MP beads are released into the environment in China^8^, up to 300 000 and 320 000 tons of MPs into North American and European farmlands, respectively^9^, while nearly 1.1 million metric tons of AFs are produced globally by domestic washing^3^. MAW is present in both marine^10^ and terrestrial^11^ environments, in urbanized^12^ and pristine^13^ ecosystems, and it can become airborne with wind, ocean surf or human assistance, to be transported to the far ends of the world^14^.

MAW is a pervasive pollutant, accumulating in both the environment and organisms^15^, with evidence of trophic transfer within aquatic^16^ and terrestrial^17^ food webs. Plants can uptake MAW through roots or leaves^18^, while humans and animals are exposed via ingestion, inhalation, and dermal contact^19^. From the human perspective, MAW has been detected in various foods, including salt, beer, milk, and bottled water^20^. Considering MPs alone, human consumption is estimated at up to 52,000 particles per year, increasing to 121,000 particles when inhalation is included^21^. In animals, MAW ingestion has been confirmed in approximately 1600 species, with around 80% being marine^22,23^, though the extent of ingestion remains under-studied. Analyses of biological samples from humans and animals have detected MPs and AFs in organs such as the brain, lungs or liver, as well as blood and breast milk^24^. Ingested or inhaled MPs and AFs can alter animal behavior^25^, cause growth reduction, inflammation, oxidative stress, biochemical and structural damage, and dysfunctions in the gastrointestinal, excretory, and reproductive systems^1^, with evidence of translocation to various organs and tissues^26^. Although the precise biological and toxicological effects are still being investigated, MAW pollution is recognized as a significant global environmental challenge.

To address MAW pollution and to estimate the risk of exposure, it is important to determine the scale of ecosystem contamination and the exposure pathway for specific groups of organisms. This has been extensively described for marine ecosystems and species, including aquatic invertebrates, fish, seabirds, sea turtles, and marine mammals^16^. In contrast, scientific attention to terrestrial ecosystems began at least a decade later^27^, although in the European Union alone the amount of plastic waste entering continental environments each year may be between 4 and 23 times the amount estimated to be released to oceans^28^. Therefore long term and large-scale monitoring data are required to assess the extent of contamination and the route of exposure in terrestrial ecosystems, as well as a basic knowledge of the scale of the problem. Wild animals can aid in this effort, as the likelihood of MAW ingestion is linked to their life history and behavior, and few sentinel species were proposed for MAW pollution monitoring^29^.

Studies on terrestrial wildlife indicate that MAW ingestion is ubiquitous. MAW may be confused with food, attached to consumed food or ingested via trophic transfer^30–32^. However, the scale, source and pathways of contamination in terrestrial ecosystems remain largely unexplored. While exposure risk is higher for species foraging in areas with increased MAW concentrations^33–35^, foraging behavior and food selection may play a significant role when considering MAW ingestion. Current field studies on terrestrial vertebrates have focused mainly on omnivorous and carnivorous species^36–41^ and less on herbivorous ones. For the latter, most analysis concerned the droppings of small mammals^31,42^ or the digestive tracts of single birds from a handful of species^43,44^. Furthermore, dietary generalists and opportunists seem to be more likely to ingest MAW, since they are less picky and consume a broad range of foods compared to specialist species, which consume a narrow range of foods, and accept only the most profitable ones^23,45^. However, the topic is still unexplored and more research is needed to fully understand the impact of MAW pollution on these species.

In this study, we investigate the ingestion of MAW in six European finch species (Passeriformes: Fringillidae; hereafter referred to as finches) and three European tit species (Passeriformes: Paridae; hereafter referred to as tits). The finch species include the Common Chaffinch (*Fringilla coelebs*), Hawfinch (*Coccothraustes coccothraustes*), European Greenfinch (*Chloris chloris*), European Goldfinch (*Carduelis carduelis*), Eurasian Bullfinch (*Pyrrhula pyrrhula*), and Eurasian Siskin (*Spinus spinus*). The tit species are the Great Tit (*Parus major*), Blue Tit (*Cyanistes caeruleus*), and Coal Tit (*Periparus ater*). These species are among the most numerous migratory passerines in Europe, primarily breeding in deciduous, mixed, and conifer forests. During the non-breeding season, finches often move to more open and urbanized areas, while tits are largely sedentary or make short-distance movements^46,47^. All the studied finch and tit species are commonly observed visiting bird feeders and have been shown to use anthropogenic materials in their nests^48^.

Finches are primarily herbivorous, feeding on seeds, buds, fruits, and flowers, even during the nestling stage, and they diversify their diet with invertebrates to varying degrees. Their specialized bill structure allows for precise seed husking. Depending on the species, they may forage on the ground or perch on thistles, grasses, and other vegetation, or scoop out cone seeds high in the tree canopy^49^. The diet of Chaffinches is the most varied among the studied finches, consisting mostly of small invertebrates, seeds, and buds, usually outside the breeding season. Other finches primarily consume plant matter such as seeds, buds, shoots, flowers, and fruit, with invertebrates added mainly during the breeding season and as food for nestlings. Chaffinches obtain most of their food from the ground throughout the year, using different vegetation layers or pursuing insects to a lesser extent. Hawfinches and Greenfinches forage roughly equally on the ground and at different vegetation layers, primarily searching for fallen seeds. Goldfinches, Bullfinches, and Siskins obtain most of their food directly from vegetation and occasionally from the ground, mainly during winter^49,50^. The studied tits are primarily insectivorous for most of the year, switching to seeds and fruits when invertebrates are scarce. They forage actively, moving in search of prey among foliage, branches, and trunks, occasionally pursuing insects in flight, and collecting fallen seeds, berries, and invertebrates on the ground^47^. Great Tits typically forage on the ground and in the lower layers of vegetation, avoiding the tallest canopies. Blue Tits forage at various levels of vegetation, while Coal Tits predominantly forage in the upper levels of conifer trees but are also frequently found foraging on the ground.

**Fig. 1:**
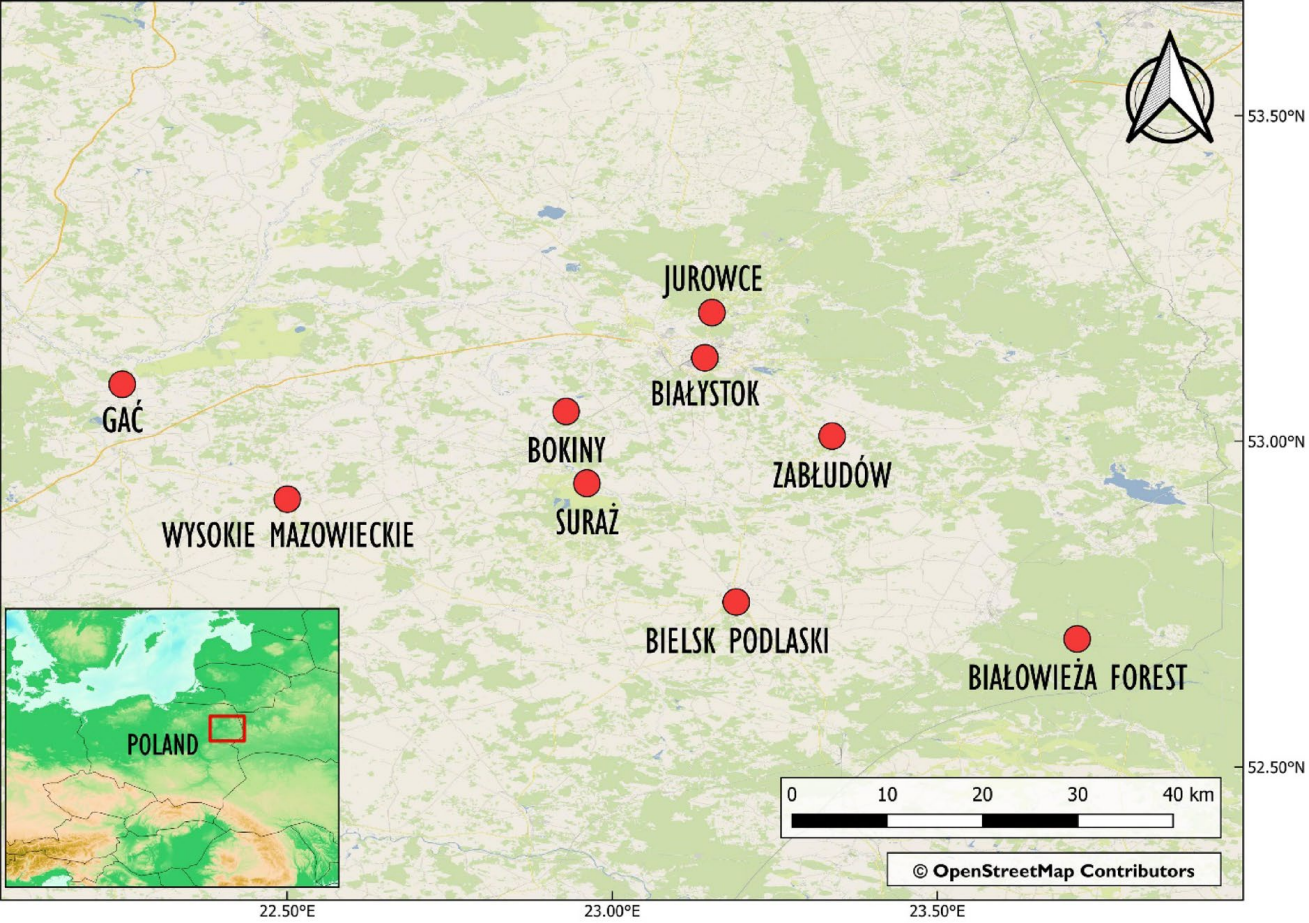
Location of bird carcass sampling sites. Detailed map generated from OpenStreetMap open data, licensed under the Open Data Commons Open Database License by the OpenStreetMap Foundation (www.openstreetmap.org). Miniature map created with GEBCO 2022 Grid (GEBCO Compilation Group). Figure created in QGIS 3.24.

The aim of the present study was to assess MAW ingestion among the selected species and to evaluate their potential as indicators for environmental pollution monitoring, given their widespread distribution beyond continental scales. Both avian families are often found in urban and agricultural areas, potentially exposing them to MAW pollution. However, because of their diet and foraging mode, which involves precision lifting of invertebrates, seeds, or plants often from the vegetation layer, we predict that the amount of MAW in the digestive tract will be low. We also looked at MAW ingestion rates in relation to the birds’ age, sex, and gastrointestinal tract section, as well as the time in season and area of carcass origin. We predict that, due to the more complex structure of the intestines compared to the stomach, the former may show higher MAW retention, indicating a propensity for MAW accumulation. Because of this, we hypothesize that older birds will have higher amounts of MAW in their digestive tracts, suggesting issues with excretion and accumulation over time. We also hypothesize that the ingestion rate of MAW will fluctuate between the breeding season (in forest habitats) and the non-breeding season (in more exposed, human-altered areas^51,52^), and predict that MAW ingestion rates will be higher during the non-breeding season when birds migrate to more human-altered areas compared to the breeding season in forest habitats (Fig. 1).

## Results

**Fig. 2:**
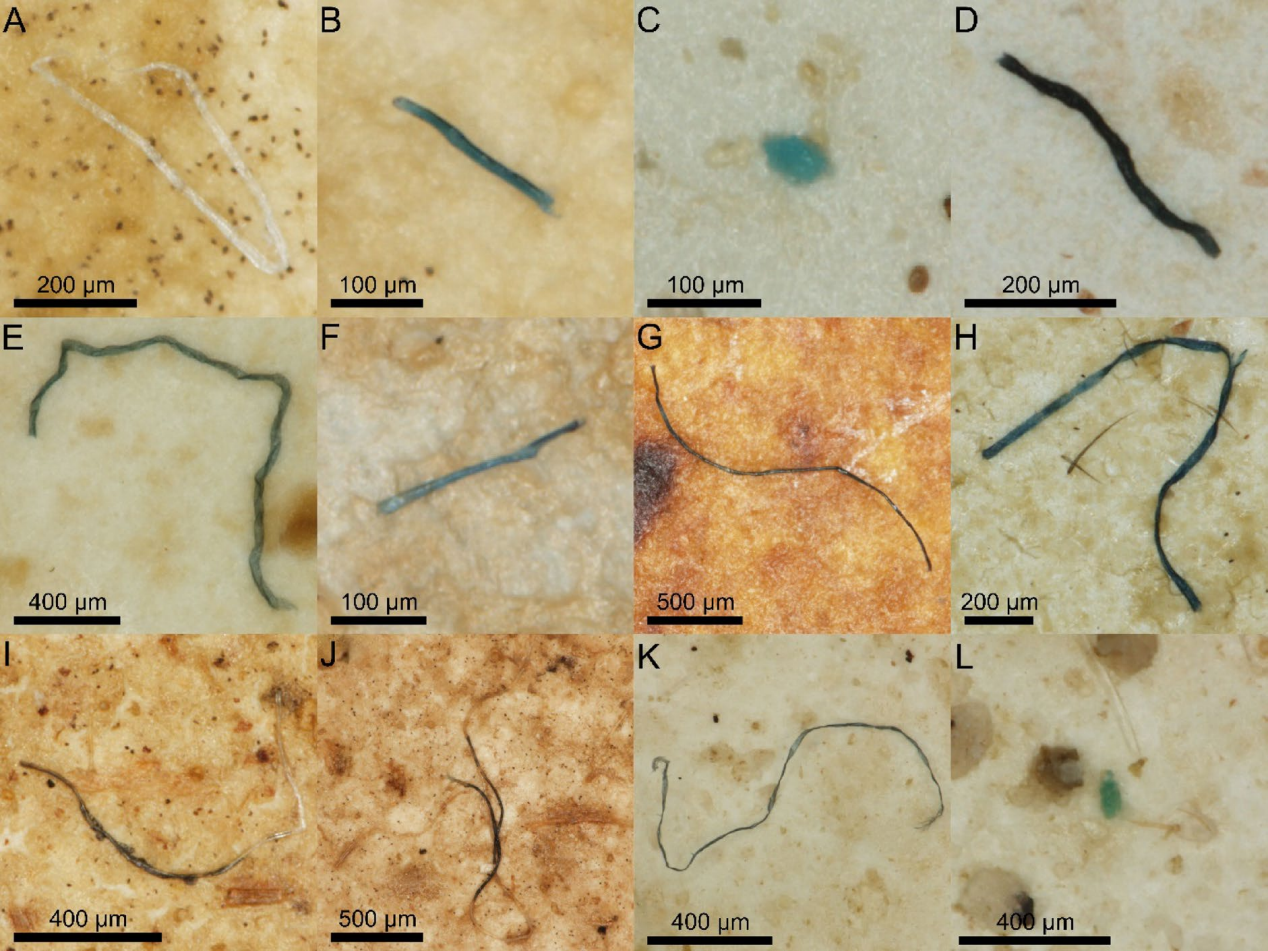
Examples of microscopic anthropogenic waste ingested by studied species. (**A**) Polyethylene fiber; (**B**) polyethylene terephthalate fiber; (**C**) polystyrene foam; (**D**) carbon nanotube fiber; (**E**, **F**) cellulose fiber; (**G**, **H**) cotton fiber; (**I**, **J**) rayon fiber; (**K**, **L**) viscose fiber.

**Fig. 3:**
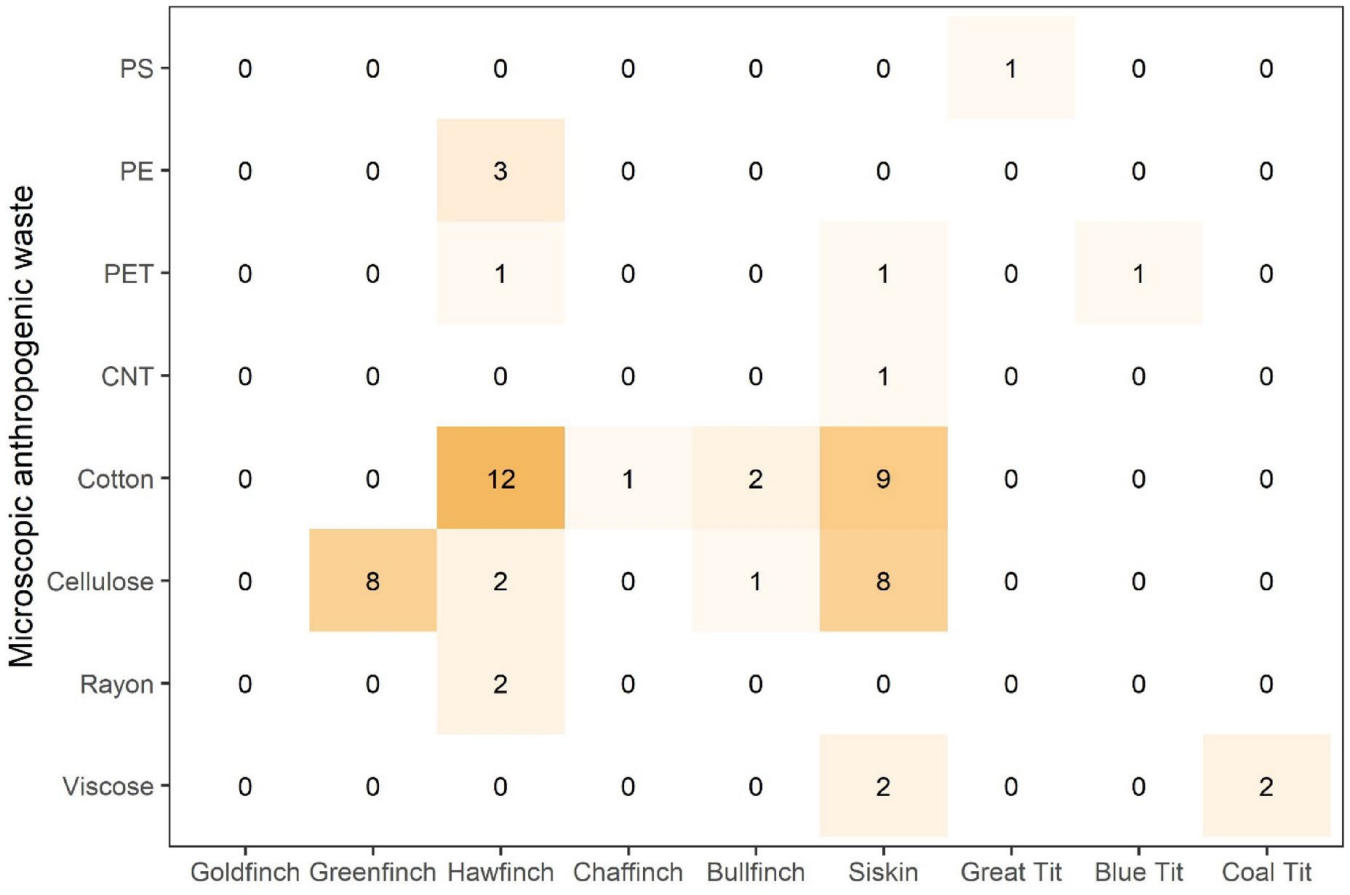
Number of microscopic anthropogenic waste particles observed in the gastrointestinal tracts of studied bird species.

Investigation of gastrointestinal tracts revealed the presence of MAW in 31 out of 149 analyzed individuals (20.8%) (Fig. 2). In total, we found 1 foam and 1 fragment classified as MPs, as well as 67 microfibers, 5 of which were classified as MPs, 1 as carbon nanotube (CNT) and 49 as AFs, of which 4 were recognized as natural cellulose and 8 as natural cotton (Fig. 3; Table A.1). Therefore, 96.5% of all observed MAW was classified as microfibers. Slightly more MAW was observed in the intestines (0.24 ± 0.60 MAW per individual) than in the stomachs (0.14 ± 0.44 MAW per individual), but these differences were not significant (Mann–Whitney U test, Z = -1.561, p = 0.122).

Raman microspectroscopy identified MPs as polyethylene (3 fibers; PE) ingested by Hawfinches, polyethylene terephthalate (2 fibers, 1 fragment; PET) ingested by Hawfinch, Siskin and Blue Tit, and polystyrene (1 foam; PS) ingested by Great Tit (Figure A.1; Figure A.2; Table A.1). One fiber ingested by Siskin was identified as carbon nanotube (Figure A.1; Figure A.2; Table A.1). AFs were identified as cellulose (19 fibers; Figure A.3), cotton (24 fibers; Figure A.4), rayon (2 fibers; Figure A.5) and viscose (4 fibers; Figure A.5) (Figure A.6; Table A.1.). We also assigned spectra of 24 fragments to quartz grains, which were ingested by Greenfinches (1 fragment), Bullfinches (1 fragment), Siskins (5 fragments) and Hawfinches (17 fragments) (Figure A.6; Figure A.7; Table A.1). Raw spectra of each type of microscopic anthropogenic waste found in the gastrointestinal tracts of studied finches and tits are available in the supplementary materials.

Mean length of all confirmed MAW was 1193.3 ± 920.3 μm (range: 88 – 4100 μm) and 82.5% of them were below 2 mm in size (Table A.2). Mean length of MPs was 510.8 ± 428.8 μm (range: 88 – 1226 μm), while that of AFs was 1304.8 ± 931.0 μm (range: 125 – 4100 μm). Mean quartz grains length was 459.3 ± 277.1 μm (Table A.2). Dominant color of observed MPs was transparent (3 fibers; 37.5%) and blue (3 fibers; 37.5%), followed by green (1 fiber; 12.5%) and black (1 fiber; 12.5%), while for AFs it was black (36 fibers; 73.5%), followed by blue (9 fibers; 18.4%), transparent (3 fibers, 6.1%) and green (1 fiber, 2.0%) (Table A.3). The color of one quartz grain was black, while the rest were transparent (Table A.3).

The highest MAW ingestion rates were observed in Siskins (0.95 ± 1.05 MAW per individual) and Hawfinches (0.91 ± 1.85 MAW per individual), followed by Greenfinches (0.53 ± 0.92 MAW per individual), Bullfinches (0.30 ± 0.48 MAW per individual), Coal Tits (0.10 ± 0.45 MAW per individual), Blue Tits (0.06 ± 0.25 MAW per individual), Great Tits (0.05 ± 0.22 MAW per individual), and Chaffinches (0.05 ± 0.22 MAW per individual). MAW ingestion was not observed in Goldfinches. The average number of MAW in the stomach was 0.14 ± 0.48 per individual, and in the intestines, it was 0.24 ± 0.66 per individual.

We fitted multiple generalized linear mixed models (GLMMs) to the data, considering various combinations of predictor variables, which resulted in a list of the top 4 models with delta AICc < 2.00 (Table 1). The model averaging process identified bird family and time in season as significant predictors of MAW ingestion, with both variables showing strong effects on the response variable (Table 2). The models revealed that bird family had a highly significant effect, indicating that finches (0.57 ± 1.12 MAW per individual) ingested significantly more MAW than tits (0.07 ± 0.32 MAW per individual). Time in season also significantly influenced ingestion levels, with higher MAW ingestion rates observed during the non-breeding period. Despite the high importance weight (1.00) of bird sex, its effect size was very small and not statistically significant in the model averaging results. Other predictors, such as bird age, gastrointestinal tract section, and site, exhibited varying levels of influence but also did not significantly impact MAW ingestion (Table 2).

**Table 1:** The table lists the top generalized linear mixed models considered in the model averaging process, ranked by their corrected Akaike Information Criterion (AICc).

Model combination	df	logLik	AICc	Delta AICc	Weight
Family + Season + Sex + GI tract section	6	− 102.56	217.55	0.00	0.44
Age + Family + Season + Sex + GI tract section	7	− 102.18	218.94	1.38	0.22
Family + Season + Sex + GI tract section + Site	7	− 102.41	219.39	1.84	0.17
Family + Season + Sex	5	− 104.56	219.42	1.87	0.17

The table includes the degrees of freedom (df), log-likelihood (logLik), AICc values, delta AICc (difference from the best model), and Akaike weights (weight), which indicate the relative support for each model given the data. Models with delta AICc < 2.00 were selected for model averaging.

**Table 2:** The table presents the model-averaged coefficients for the predictors included in the top generalized linear mixed models, based on their Akaike weights.

Predictor	Estimate	Std. error	Z value	Weight	*P*
(Intercept)	− 2.635	1.532	1.711		0.087
Family	− 1.804	0.770	2.328	1.00	**0.020**
Age	− 0.416	0.477	0.873	0.22	0.383
Sex	− 0.003	0.484	0.006	1.00	0.996
Gastrointestinal tract section	0.624	0.320	1.939	0.83	0.053
Season	1.063	0.532	1.987	1.00	**0.047**
Site	0.283	0.512	0.550	0.17	0.582

The models were averaged based on their Akaike weights, considering models with delta AICc < 2.00. The estimates are shown along with their adjusted standard errors, z-values, and p-values. Weight indicates the relative importance of predictor variables in model averaging process. Significant predictors are indicated in bold.

## Discussion

For the first time, our study determined the ingestion of MAW by nine common European passerine species from the finch and tit families. As anticipated, the ingestion rate of MAW was very low, even when compared to other passerine studies. For example, the average MPs concentration in the digestive tracts of migratory passerines in the Midwest USA exceeded 20 MPs^44^. In contrast, 2 MPs per specimen were observed in the stomach flush of Seaside Sparrows (*Ammospiza maritima*) from the Mississippi Gulf Coast^53^, and two thrush species from northeastern Poland ingested an average of 31 MPs per individual^38^. However, the latter two studies did not use analytical methods to chemically confirm the samples. Additionally, MPs and AFs concentrations were found to be lower in finches and tits, which are potential prey for birds of prey such as Northern Goshawks (*Accipiter gentilis*) and Eurasian Sparrowhawks (*Accipiter nisus*), with 6.6 MPs/specimen and 6.2 MPs/specimen, respectively^41^. This may suggest biomagnification of MPs and AFs pollution along the food web. Small passerine birds like finches and tits play a crucial role in food webs and may act as vectors of plastic between different trophic levels^54^. Given their predominantly herbivorous and insectivorous diet, they may facilitate MAW transport to higher trophic levels from soil (when feeding on the ground), plants^55^ and invertebrates, as their potential prey are active MAW samplers, as shown for honeybees^56^ and ants^32^. However, our results suggest that the scale of this process may not be significant.

Most of the observed MAW was classified as fibers, which are recognized as the most abundant form of MAW in the environment^3^. Raman analysis results indicate that MPs are infrequently ingested by finches, comprising only 14.0% of all observed MAW. This aligns with previous studies showing that MPs constitute only a small fraction of all observed microfibers, with AFs being more commonly reported in the environment. However, projections suggest that the proportions of MPs and AFs in the environment may reverse within this decade due to the increasing use of plastics in daily life^6^.

Among the MPs, PE was the most common polymer type, followed by PET and PS. PE, PET and PS are used in various industries due to their properties, such as flexibility, lightness, and chemical and thermal resistance, which make them valuable materials for a wide range of applications like packaging, food storage or fibers for textile production. PET, PE and PS are also among the most used polymers in European agriculture^57,58^. PET and PE were the most commonly identified types of polymers in the gastrointestinal tracts of North American migratory birds^44^ and birds of prey from central Spain^41^, and were observed in the faecal sacs of Tree Swallow (*Tachicyneta bicolor*) nestlings in southern Ontario, Canada^49^. Moreover, PET was the most frequently confirmed MP in Barn Owl (*Tyto alba*) pellets from northern Italy^59^, and the gastrointestinal tracts of birds of prey from central Florida, USA^36^. The ingestion of PS by birds appears to be uncommon, having not previously been documented in passerines, while reported for gulls and storks^60^.

CNT has not yet been found among the types of MAW ingested by wild birds or other terrestrial species from higher trophic levels^61^. CNT are a class of engineered nanoparticles, which due to their physical and chemical properties are used in various consumer, industry or medical applications^62^, such as coatings for cotton fabrics^63^ or sponges applied in environmental cleanup such as water purification and during oil spills^64^. The toxicity and risk assessment of CNT has been explored on marine and terrestrial organisms^61,62^. Recent studies showed that CNT induces biochemical and histopathological changes, increases the parasite load in (*Lumbricus terrestris*) earthworms^65^ and causes genotoxicity, inflammation and oxidative stress in mammals^66^. With the rapid growth of CNT production, the number of issued patents and increased market value^67^, wildlife exposure to ingestion or inhalation of this material will likely increase.

We classified 49 fibers as man-made AFs while 12 fibers were recognized to be of natural origin, as they did not provide characteristic spectral information for dyed fibers and were transparent in color. However, a few cellulose and cotton AFs in which Raman analysis showed the presence of dyes or other artificial additives were of transparent color as well, or were partially black and transparent, which may suggest dye washout during sample preparation, ingestion by the bird or earlier. Signals of dye or other additives are usually most prominent in the Raman spectrum, making Raman analysis crucial for proper identification of the AFs^68^. Confirmed AFs were classified as cellulose, cotton, rayon, and viscose, likely derived from textile materials. These fibers entered the environment through natural wear and tear, fragmentation of larger textile products, or various stages of the textile processing industry. The origin of ingested AFs may be similar, as there were no significant differences in their color.

The ingestion of AFs by animals remains under-reported, partly due to the predominant scientific focus on MPs pollution. Research has documented the ingestion of artificial cellulose, cotton, and other fibers by fish^69^ and seagrass macrofauna^70^ in marine environments. In marine birds, a high proportion of AFs was observed in King Penguin (*Aptenodytes patagonicus*) faecal samples, with cellulose, cotton, wool, and viscose comprising 88% of all analyzed samples^71^. In terrestrial environments, anthropogenic cellulose was the most prevalent polymer found in birds of prey in Florida, USA^36^, and Tree Swallows in Ontario, Canada^40^. Birds of prey from central Spain also ingested and inhaled more AFs than MPs, with proportions even more striking than those observed in our finches (Wayman et al. 2024). Natural fibers were also detected in several bird species from Shanghai, China, although their identification was based on a melting test without chemical confirmation^43^.

The global production of cellulose-based textiles continues to grow, with projections estimating an increase to 133.5 million tons by 2030^72^. The ingestion, inhalation, or skin contact with AFs poses significant health risks to both wildlife and humans. While ingestion of AFs is relatively low, inhalation remains a primary exposure route for humans and other organisms^73^. Inhalation of AFs can result in allergies, lung inflammation, and irreversible obstructive lung diseases^3^. Ingested microfibers can irritate the digestive tract, particularly if they are small, persist in the body for extended periods, and release dyes, chemical finishing agents, and adsorbed environmental pollutants. These factors can lead to various health issues, ranging from oxidative stress to cancer^6^. Therefore, further research is essential to better understand the extent of AF contamination in the environment and the likelihood of their ingestion and inhalation by living organisms.

Several fragments observed in the stomachs and intestines of Greenfinch, Hawfinch, Bullfinch, and Siskin were identified as quartz, a sand-forming mineral that may function as gastroliths. Birds ingest gastroliths to aid in breaking down hard food items like seeds^74^, which may also contribute to the fragmentation of ingested MAW. While most quartz fragments were roughly circular, some were flatter and resembled broken glass, potentially originating from laboratory glassware used during the study. Although no glass fragments were found in the blank samples, we still considered them as contamination introduced from the laboratory glassware. The presence of quartz fragments highlights the challenges of visual inspection, which can lead to false positives regarding the occurrence of microplastics, as quartz can resemble plastic fragments. Therefore, chemical analysis is necessary for accurate identification^75^.

Significant differences in MAW ingestion rates were observed among the studied bird families, with finches ingesting considerably more than tits. These differences may be related to their food preferences and foraging locations. While tits are more insectivorous and typically select their food from various layers of vegetation, finches often forage for seeds that have fallen to the ground, where anthropogenic waste accumulates the most. Vegetation can capture some microplastics through leaf surfaces and stems. However, the structure of vegetation does not effectively retain microplastics, especially when considering factors like wind and rain that can dislodge particles from plant surfaces. The ground acts as a primary sink for these particles, resulting in higher concentrations compared to vegetation layers^1,9,11^.

The lack of significant differences between the stomach and intestines, and between younger and older individuals, suggests that MAW does not accumulate in the gastrointestinal tracts of finches and tits but is gradually excreted back into the environment. However, while the difference in MAW accumulation between the stomach and intestines was not statistically significant, our results indicate a potential trend that may warrant further investigation with a larger sample size or additional data. The passage of food through the digestive tract of passerine birds occurs within hours^76^, but larger plastic debris in seabirds can reside for several days to weeks^77^. This indicates that MAW consumption may be accidental and influenced by factors such as recent contamination of feeding sites or the residence time of MAW in the digestive system.

Our data does not indicate a site-related difference in MAW ingestion rates between urban and rural areas where the carcasses were sampled. For instance, the most widespread and common ground-feeding urban dwellers with the least specialized diets, the Chaffinch and Great Tit, ingested only one AF and one MP, respectively. However, due to the limited sample size and birds’ mobility, a definite conclusion is difficult. Birds can move freely between different habitats within a brief time, and were shown to be a vector of plastic between different environments^54^. Studies suggest higher atmospheric deposition of MAW in urban areas^78^ and greater plastic accumulation in urbanized regions^79^. Analyzing MAW site contamination or atmospheric fallout would be beneficial, but was not part of this study. Nevertheless, our data indicates that adult finches and tits may not be a good MAW pollution indicator, and more reliable data might originate from the analysis of the faecal sacs of their nestlings^29,40^.

Interestingly, we observed significantly higher MAW ingestion rate by studied species during non-breeding season. The observed difference may be due to the fact that, after the breeding season, these birds usually migrate from nesting forest habitats to more open and transformed ones (e.g., meadows, pastures, agricultural fields, residential areas, or allotments) in search of food, as well as a diet switch from more animal-based to plant-based food^46,47,49^. MPs abundance in forest habitats was shown to be higher with increasing anthropopressure^52^, but significantly lower when compared to urban or agricultural areas^51^. Moreover, agricultural ecosystems, widely used by the studied finches during their non-breeding period, were listed as those with significant MAW pressure^18,55^, characterized by higher MPs’ abundance in winter than spring^80^. Such a difference is explained by more intensive agricultural activities during and after harvest, which introduce new MAW into the soil^81^, increased MPs’ degradation due to higher temperatures and UV radiation during the warmer seasons^27^ and higher deposition of airborne MAW into the soil caused by rainfall^82^, which for Poland is more intense in the summer and autumn months than in winter and spring. In the case of tits, during the non-breeding season they generally observed in wider range of habitats with varying levels of anthropopressure^47^. Moreover, during the non-breeding season, studied species reduce the proportion of animal food in their diet and rely more on plants and seeds, which they often seek on the ground or in bird feeders. This suggest that finches and tits may be less vulnerable to MAW contamination in breeding habitats, with the likelihood of consumption of MAW possibly increasing post breeding, during migration to more open and human-transformed environments.

In conclusion, our research is the first to demonstrate that common European finches and tits ingest MAW, albeit in small quantities, and do not seem to retain it in their gastrointestinal tracts. Consequently, they may not be suitable candidates as indicator species for MAW pollution. However, our study highlights the feasibility and advisability of using birds that have died from collisions with anthropogenic surfaces for analyzing MAW ingestion. Future research should focus on MAW consumption by nestlings to better understand the vulnerability of finches, tits, and other small terrestrial avian species to MAW pollution on a broader scale.

## Methods

### Sampling

Bird carcasses were collected during wildlife monitoring in the anthropogenic infrastructure of the Podlaskie Voivodeship in north-eastern Poland, between 2019 and 2023 (Fig. 1). The collected birds had died as a result of collisions with transparent road acoustic screens and the glazed facades of buildings in Białystok (N53.1084, E23.1542) and Jurowce (N53.1966, E23.1556) (urban sites), collisions with high voltage powerlines near Suraż (N52.9490, E22.9564) and Gać (N53.0806, E22.2463) (rural sites), and collisions with cars in Białowieża Primeval Forest (N52.7055, E23.7168) and near Zabłudów (N53.0145, E23.3384), Bielsk Podlaski (N52.7654, E23.1864), Bokiny (N53.0422, E22.9172), and Wysokie Mazowieckie (N52.9152, E22.5096) (rural sites). A total of 22 Hawfinches, 22 Siskins, 20 Chaffinches, 15 Greenfinches, 10 Bullfinches, 4 Goldfinches, 20 Great Tits, 16 Blue Tits and 20 Coal Tits from seven sampling sites were used in the current study (Table A.1). The collected carcasses were fresh, with complete feathering and without external wounds on the body. They were put in individual bags, frozen within 12 h of collection and stored at − 20 °C until further analysis. Carcass collection and storage was carried out with permission from the Regional Directorate of Environmental Protection in Białystok to KD (WPN.6401.174.1.2019.MC) and SN (WPN.6401.41.2019.MC).

### Contamination prevention

To prevent sample contamination during laboratory analysis quality assurance and quality control guidelines during the preparation, quantification, and characterization of MAW were followed^83^. Laboratory work was carried out under a chemical laminar hood in a closed laboratory room with restricted access. We used only glass and metal instruments, which were cleaned three times before every specimen analysis, with demineralized water filtered through Millipore 0.2 μm PC 47 mm black membrane filters. We used the potassium hydroxide solution (KOH) digestion technique to dissolve birds’ gastrointestinal tracts, as it is a recommended extraction method for MAW exposure risk studies^36,84^. The 10% KOH solution used to dissolve the bird’s stomach and intestines was filtered through Millipore 0.45 μm PTFE 47 mm black membrane filters. Before moving the stomach and intestines into a glass test tube with KOH, they were flushed with filtered water to remove blood, feathers and any external contaminants which may have reached their surface during dissection. Prior to filtration, each filter was examined to detect contamination from external sources. Finally, we performed 20 laboratory blank tests for potential airborne contamination, which were treated and analyzed in the same way as stomach and intestine samples. Blank tests showed no MAW or quartz contamination.

### Preparation, quantification and characterization of MP

Before dissection, specimens were stored at room temperature for 6 h to defrost. Afterwards, all carcasses were aged by plumage characteristics^85^. Bird ages were divided into two categories: 1—hatched during the year when the carcass was found; 2—hatched one or more years before the carcass was found. Considering the period in the year in which the carcass was found and the breeding biology of the studied species, we classified carcasses found from April to August as during the breeding season, and those that died during other months as non-breeding.

During dissection, a complete digestive tract was removed from each carcass. A section including the proventriculus and gizzard with 1 cm of gastrointestinal tract on both sides (referred to further as the stomach) and small and large intestine (referred to further as intestines) was sampled for further analysis. Stomachs and intestines were cleaned with filtered demineralized water, transferred to a glass test tube, and submerged in 10% KOH solution. Test tubes were placed in a heated bath set to 55 °C and left for 72 h, allowing the organic material to dissolve completely. Subsequently, the contents of the test tube were filtered in a vacuum filtration system using Millipore 0.45 μm PTFE 47 mm membrane filters. The filters were then moved to glass petri dishes with glass covers and left to dry under the chemical laminar hood. Finally, they were sealed and moved to a refrigerator and stored at 2° C until further analysis.

Filters were visually inspected under 72 × magnification using an Olympus DSX110 inverted microscope. To monitor potential airborne cross-contamination, each filter was inspected under 12 × magnification prior to the actual analysis. We took into consideration MAW of sizes ranging between 80 μm and 5 mm. The upper size limit is the standard adopted in MPs studies, while the lower size limit indicates particles which we could effectively move with a steel preparation needle and subject to the sample purification process. We identified the color and measured the length (along the longest surface) of each piece of MAW observed.

We quantified the amount of MAW and identified its type using Raman microspectroscopy, which enables the identification of MPs, AFs, and various additives with spatially resolved detection reaching the limit of 1 µm^68,86^. To prevent fluorescence, sample purification was applied before Raman analysis. Enzymatic treatments were applied to particles, because proteins show a natural glow due to the presence of fluorophores^44^. Each sample was incubated for 60 min at 52° C in Proteinase-K solution to denature proteins^84,87^. Afterwards, samples were rinsed in filtered demineralized water, dried, placed on gold coated microscope slides and moved to a Raman microscope chamber. Raman spectra were recorded using a Renishaw InVia Raman microscope equipped with a thermoelectrically (TE)-cooled CCD detector and semiconductor laser, emitting light at 785 nm wavelengths. The spectra were recorded in the 100 − 3200 cm^−1^ spectral range with a resolution higher than 2 cm^−1^. The laser beam focused on the sample with the objective × 20 and × 50 was kept at about 1 mW power. This was selected to avoid sample damage. The signals were recorded using a Peltier-cooled charge-coupled device (CCD). Spectra were acquired with 10 accumulations, while the most suitable scanning times were determined experimentally for each sample separately, as well as the laser’s power to receive spectra of the best quality. A sample Raman spectra was evaluated using a correlation chart of characteristic group frequencies and KnowItAll Software and IR & Raman Spectral Library (John Wiley & Sons, Hoboken Inc.). Given that finches are herbivorous, and cotton and cellulose can have either an anthropogenic or natural origin, we used only colored AFs, confirmed by Raman spectra in the analysis, and we excluded clear ones^6^.

### Statistical analysis

We employed Generalized Linear Mixed Models (GLMMs) to analyze the factors influencing the ingestion of MAW among different bird species. The primary model included predictor variables such as bird family (finches vs tits), sex, season (breeding vs non-breeding), gastrointestinal tract section (stomach vs intestines), and site type (urban vs rural). Bird ID was included as a random effect to account for individual variability. GLMMs were fitted using the glmer function from the lme4 package^88^, with a Poisson distribution and log link function. All possible combinations of the predictor variables were considered. To ensure the robustness of our model, we calculated the corrected Akaike Information Criterion (AICc) for all possible combinations of predictor variables. Models with a delta AICc < 2.00 were selected for model averaging^89^. Model averaging was performed using the MuMIn package^90^ to obtain averaged coefficients, standard errors, and significance levels. To assess the relative importance of predictor variables, we used the sw function from the MuMIn package^90^. This function sums the Akaike weights (AIC weights) for each variable across all models in which it appears, providing a measure of each variable’s contribution to the model’s predictive power. Overdispersion was checked by comparing the residual deviance to the degrees of freedom, confirming that the Poisson regression model was appropriate for our data. The analyses were conducted in R Studio^91^. Data are presented as means ± SD unless otherwise specified.

## Electronic supplementary material

Below is the link to the electronic supplementary material.

Supplementary Material 1

Supplementary Material 2

## Data Availability

The datasets generated during and/or analysed during the current study are available from the corresponding author on reasonable request.
