# Supplementary material for: Microscopic anthropogenic waste ingestion by small terrestrial European passerines: evidence from finch and tit families
Source: Sci Rep. 2025 May 13;15:16631. doi: 10.1038/s41598-025-01608-9 (PMC12075826; doi:10.1038/s41598-025-01608-9)
Supplement: Supplementary file 2 — Supplementary Material 2 [file 41598_2025_1608_MOESM2_ESM.doc]

**Microscopic anthropogenic waste ingestion by small terrestrial European passerines: evidence from finch and tit families**

Krzysztof Deoniziak^a^*, Anna Winiewicz^b^, Marta Nartowicz^b^, Weronika Mierzejewska^b^, Sławomir Niedźwiecki^c^, Wojciech Pol^d^, Alina T. Dubis^e^

^a^ Division of Biodiversity and Behavioural Ecology, Faculty of Biology, University of Bialystok, Konstantego Ciołkowskiego 1J, 15-245 Białystok, Poland

^b^ The Włodzimierz Chętnicki Biological Science Club, Faculty of Biology, University of Bialystok, Konstantego Ciołkowskiego 1J, 15-245 Białystok, Poland

^c^ Glass Traps Foundation, Brzezina 10, 55-330 Brzezina, Poland

^d^ Department of Water Ecology, Faculty of Biology, University of Bialystok, Ciołkowskiego 1J, 15-245 Białystok, Poland

^e^ Faculty of Chemistry, University of Bialystok, Konstantego Ciołkowskiego 1K, 15-245, Białystok, Poland

*Corresponding author email address: [k.deoniziak@uwb.edu.pl](mailto:k.deoniziak@uwb.edu.pl)


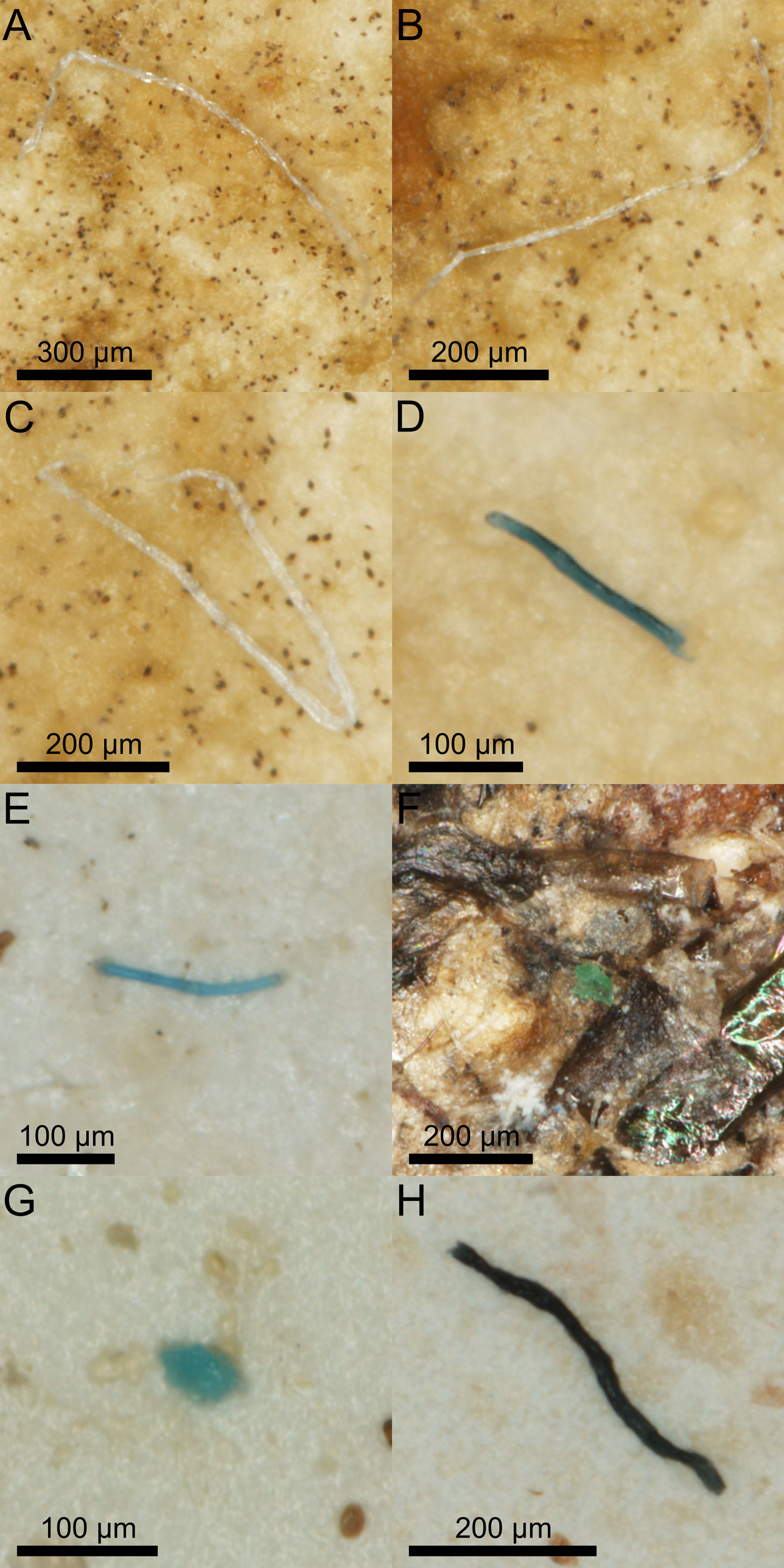


**Figure A.1.** Examples of microscopic anthropogenic waste found in the stomach and intestines of studied species. A, B, C – transparent polyethylene (PE) fiber; D, E – blue polyethylene terephthalate (PET) fiber; F – green polyethylene terephthalate (PET) fragment; G – blue polystyrene (PS) foam; H – black carbon nanotube (CNT) fiber.


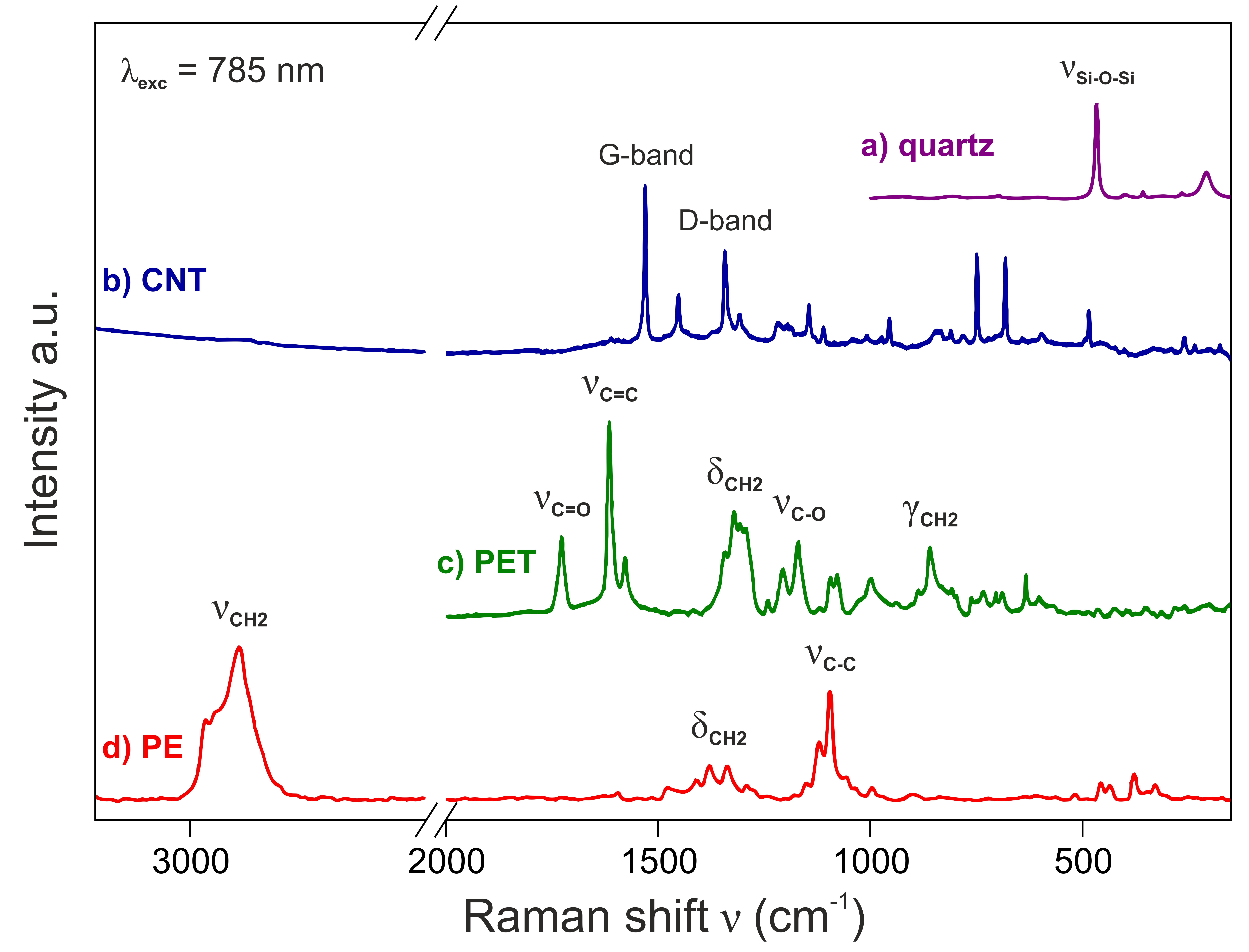


**Figure A.2**. Raman spectra of observed anthropogenic microscopic litter and quartz fragments. a – quartz; b – carbon nanotube (CNT); c – polyethylene terephthalate (PET); d – polyethylene (PE). Figure 1b shows a spectrum with two prominent Raman bands at 1530 and 1343 cm^-1^, which could be assigned to the G and D bands of C-C stretching vibration of CNT. Figure 1c shows the PET spectrum, where the most prominent Raman bands in 1727 and 1615 cm^-1^ were assigned to carbonyl (C=O) groups and C=C double bonds. Figure 1d presents Raman bands at 2897, 1470, 1379, 1120, and 1095 cm^-1^, which correspond to the stretching and bending vibration of the CH_2_ groups and the C-C stretching vibration of the polymer chains, close to the PE spectrum.


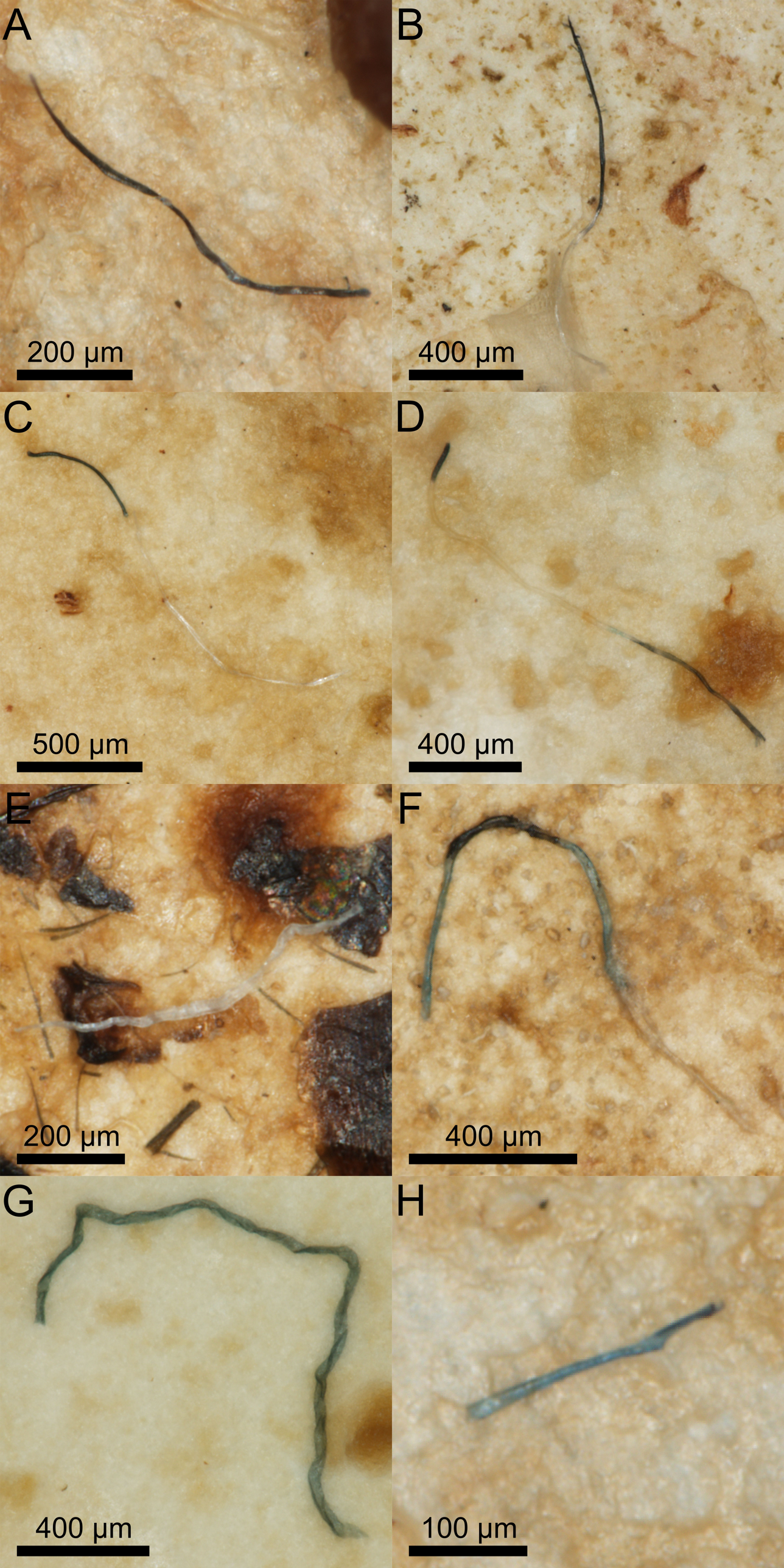


**Figure A.3.** Examples of cellulose fibers found in the stomach and intestines of studied species.


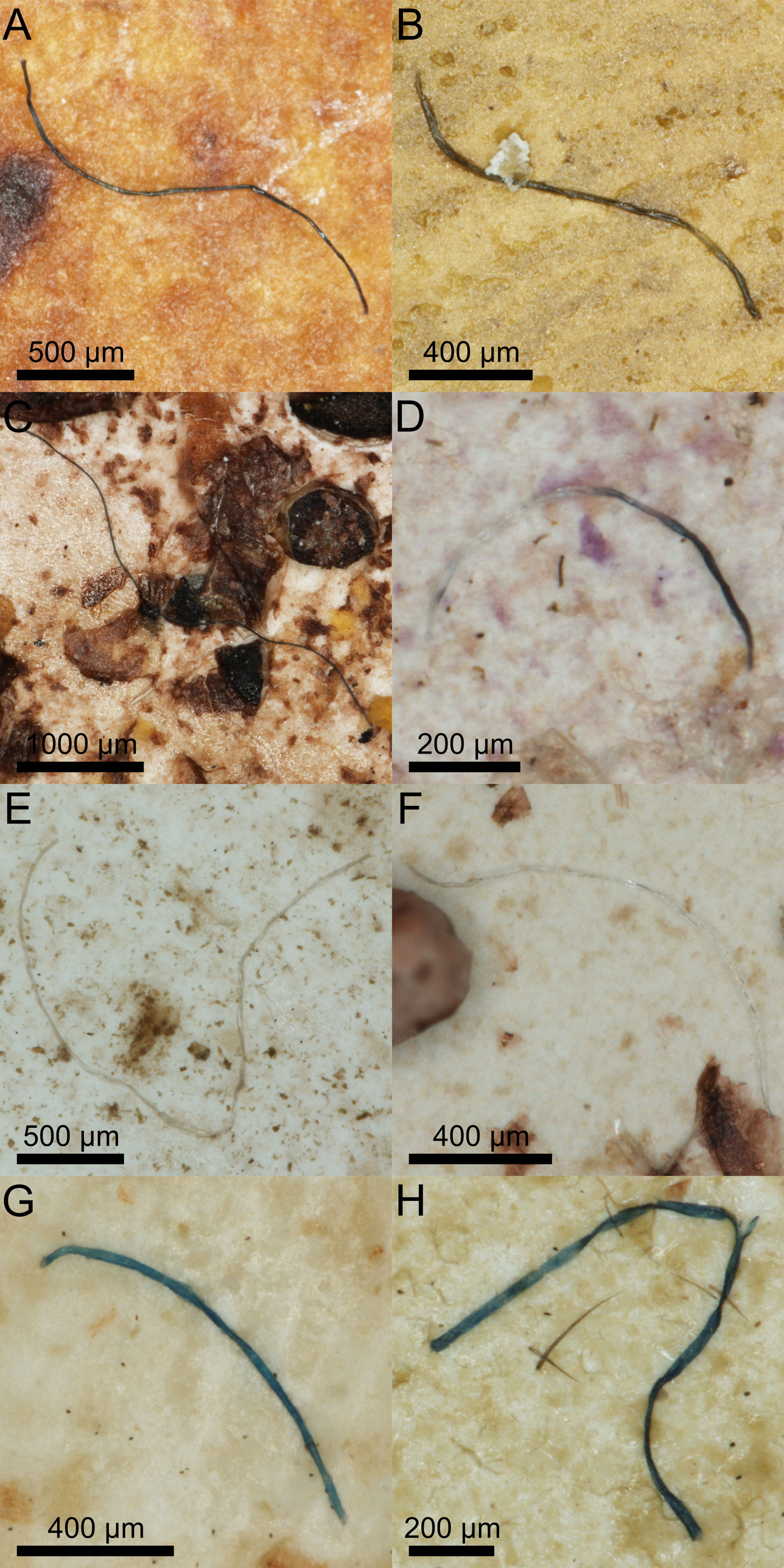


**Figure A.4.** Examples of cotton fibers found in the stomach and intestines of studied species.


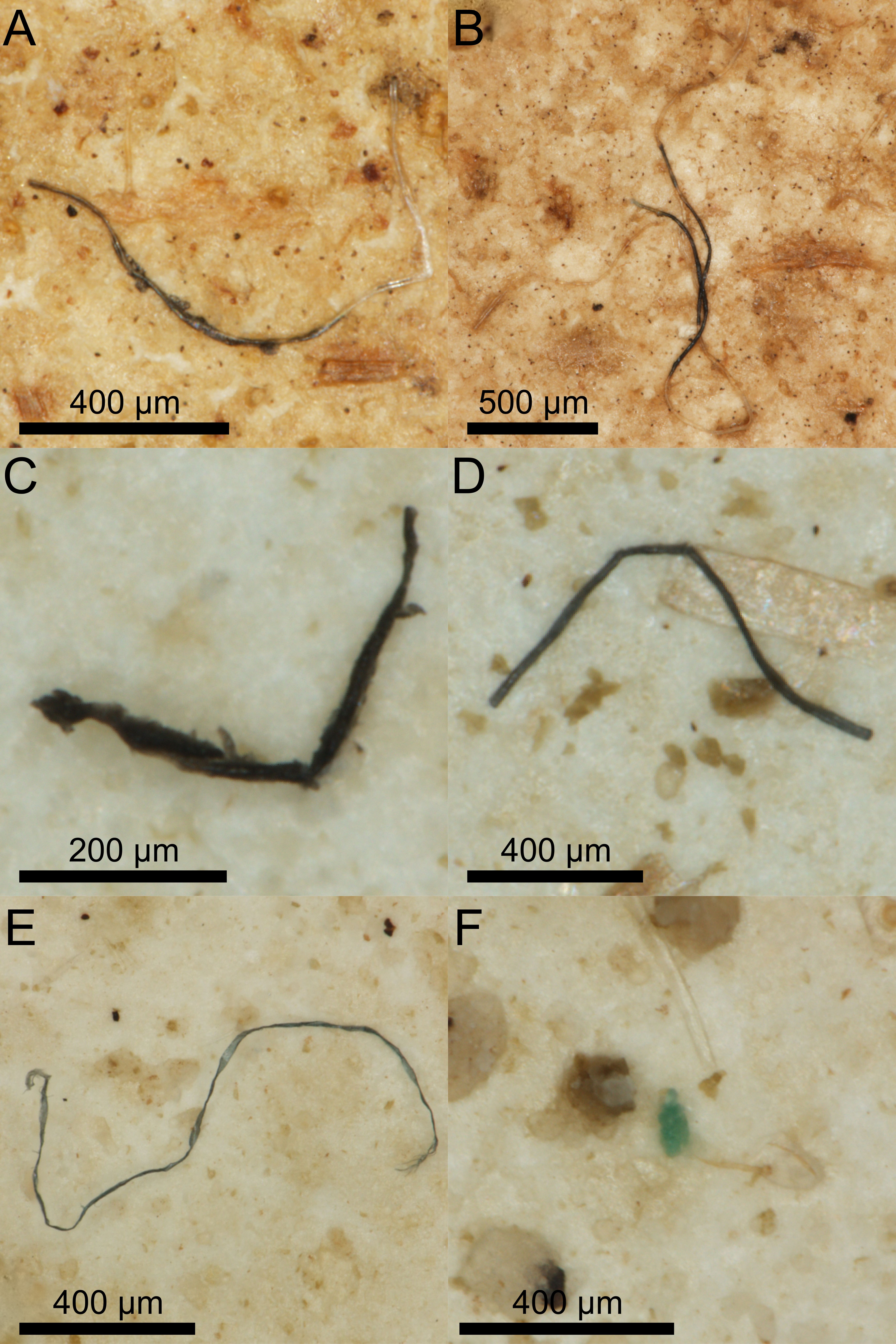


**Figure A.5.** Rayon (A, B) and viscose (C, D, E, F) fibers found in the stomach and intestines of studied species.


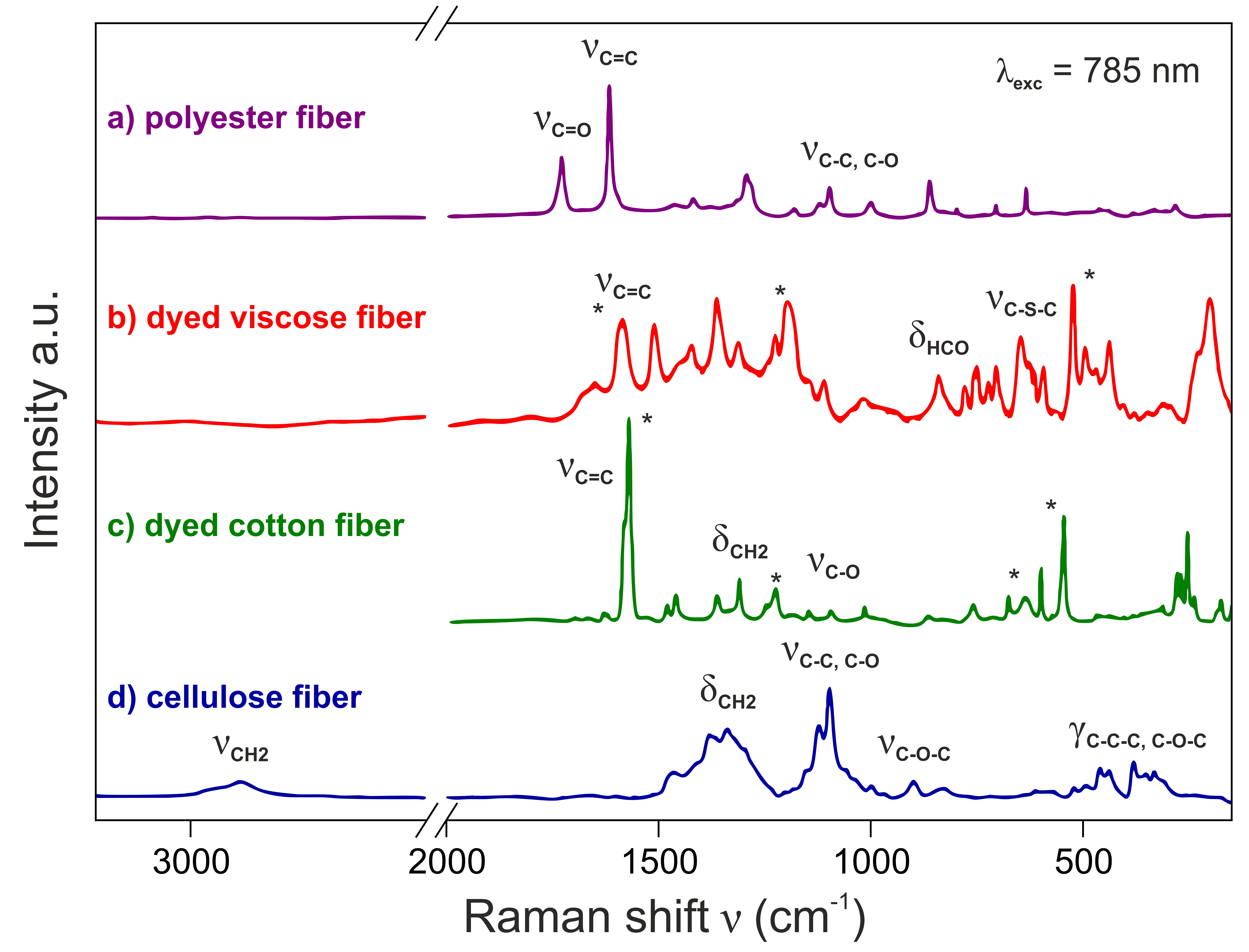


**Figure A.6.** Raman spectra of selected microfibers. a – polyester fiber; b – dyed viscose fiber; c – dyed cotton fiber; d – cellulose fiber. Asterisks indicate bands coming from the dye. Figure 2a represents the spectrum of a polyester fiber used as a reference sample, which shows a medium intense carbonyl (C=O) band at 1728 cm^-1^, a very intense band at 1616 cm^-1^ due to the stretching vibration of C-C of aromatic rings, and other characteristic bands at 1293 cm^-1^ due to COO and at 1097 cm^-1^ due to C-O and C-C vibrations. The spectra in Figure 2b and Figure 2c were assigned to dyed viscose and cotton fibers, respectively. The Raman spectrum of viscose and cotton are similar since they are close in structure, while the unique feature of the viscose Raman spectrum is a peak at around 650 cm^-1^ of the stretching vibration of C-S-C bonds. The cellulose spectrum in Figure 2d reveals a broad band with a maximum at 2895 cm^-1^ due to the stretching and deformational vibration of CH_2_ groups and the most characteristic signal at 1152, 1123, and 1097 cm^-1^ due to C-C ring stretching and C-O-C glycoside bonds stretching.


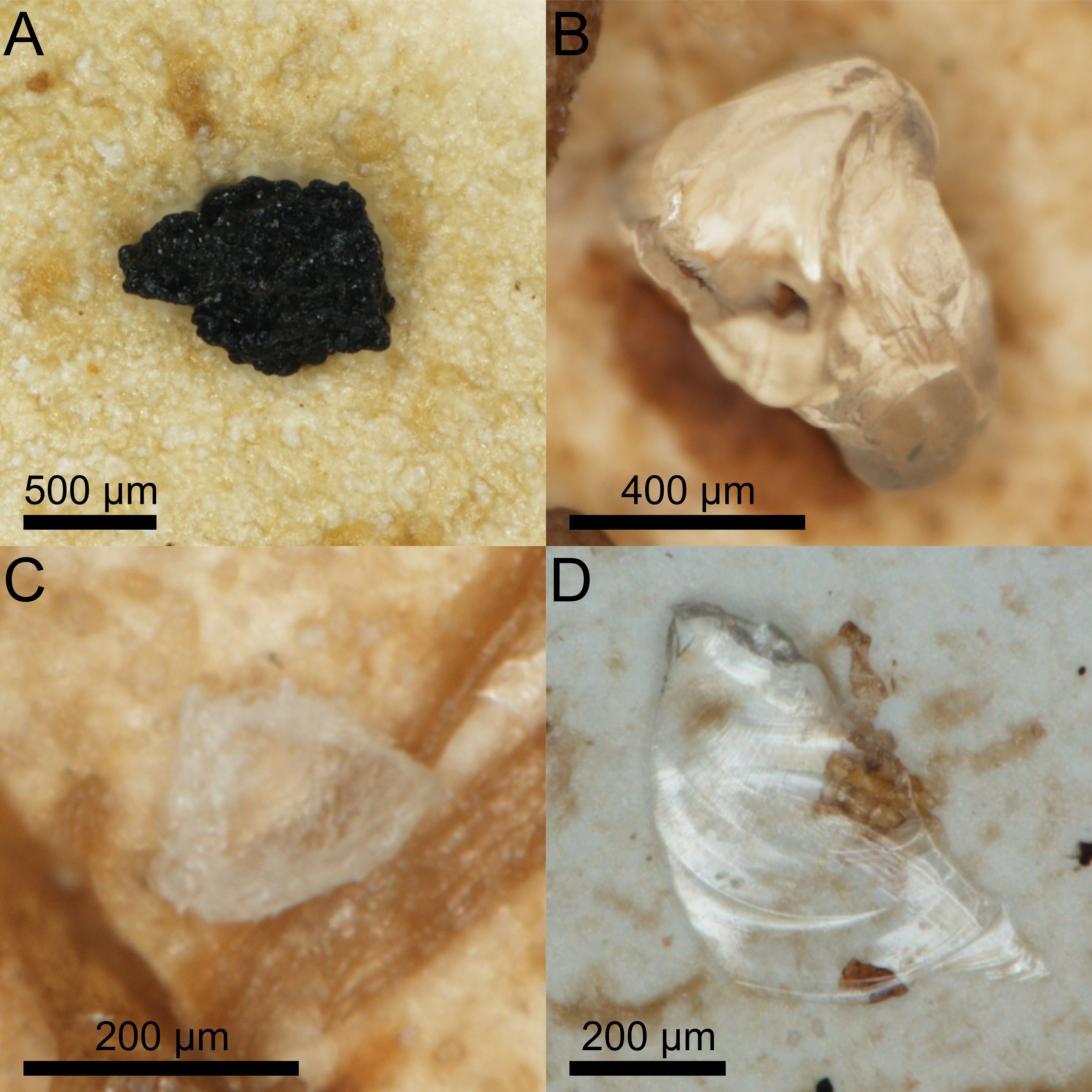


**Figure A.7.** Examples of SiO_2_ fragments found in the stomach and intestines of studied species.

**Table A.1.** Age, sex, sampling date, sampling location and number of anthropogenic litter found in stomach and intestines of analyzed individuals. Species codes: CA - European Goldfinch; CH – European Greenfinch; CO – Hawfinch; FR – Eurasian Chaffinch; SP – Eurasian siskin; PY – Eurasian bullfinch; PM – Great Tit; CC – Blue Tit; PA – Coal Tit. Litter codes: SiO_2_ – sand grain (quartz); Ce – cellulose; DCe – dyed cellulose; Co – cotton; DCo – dyed cotton; Ra – rayon; VI – viscose; CNT – carbon nanotubes; PET – polyethylene terephthalate; PE – polyethylene; PS - polystyrene

| **ID** | **Age** | **Sex** | **Date** | **Location** | **Sample** | **SiO2** | **Ce** | **DCe** | **Co** | **DCo** | **Ra** | **Vi** | **CNT** | **PET** | **PE** | **PS** |
| --- | --- | --- | --- | --- | --- | --- | --- | --- | --- | --- | --- | --- | --- | --- | --- | --- |
| CH08 | 2 | F | April 2020 | Jurowce | Stomach | 1 |  |  |  |  |  |  |  |  |  |  |
| CH09 | 1 | F | December 2019 | Białystok | Stomach |  |  | 1 |  |  |  |  |  |  |  |  |
| CH10 | 2 | M | November 2019 | Białystok | Intestines |  |  | 1 |  |  |  |  |  |  |  |  |
| CH11 | 1 | M | January 2020 | Białystok | Stomach |  |  | 1 |  |  |  |  |  |  |  |  |
| CH12 | 1 | F | March 2020 | Jurowce | Intestines |  |  | 2 |  |  |  |  |  |  |  |  |
| CH13 | 2 | F | December 2019 | Białystok | Stomach |  |  | 2 |  |  |  |  |  |  |  |  |
|  |  |  |  |  | Intestines |  |  | 1 |  |  |  |  |  |  |  |  |
| CO01 | 1 | F | August 2022 | Białystok | Stomach | 4 |  |  |  |  |  |  |  |  |  |  |
| CO03 | 2 | F | September 2022 | Białystok | Intestines |  |  |  |  |  |  |  |  |  | 3 |  |
| CO04 | 1 | **-** | September 2022 | Białystok | Stomach | 1 |  |  |  | 2 |  |  |  |  |  |  |
|  |  |  |  |  | Intestines | 2 |  |  |  | 5 |  |  |  |  |  |  |
| CO06 | 1 | M | August 2022 | Białystok | Stomach |  |  | 1 |  | 2 |  |  |  |  |  |  |
|  |  |  |  |  | Intestines |  |  | 1 |  |  |  |  |  | 1 |  |  |
| CO09 | 1 | M | July 2022 | Białystok | Intestines | 2 |  |  | 1 | 1 |  |  |  |  |  |  |
| CO11 | 1 | **-** | August 2022 | Białystok | Stomach | 4 |  |  |  |  | 1 |  |  |  |  |  |
|  |  |  |  |  | Intestines | 4 |  |  |  |  | 1 |  |  |  |  |  |
| CO13 | 2 | M | October 2021 | Białystok | Stomach |  |  |  |  | 1 |  |  |  |  |  |  |
| CO14 | 1 | F | September 2022 | Białystok | Intestines |  |  |  | 2 |  |  |  |  |  |  |  |
| CO18 | 2 | M | August 2022 | Białystok | Stomach |  |  |  |  | 1 |  |  |  |  |  |  |
| FR03 | 2 | M | April 2020 | Białowieża | Stomach |  | 2 |  |  |  |  |  |  |  |  |  |
|  |  |  |  |  | Intestines |  |  |  |  | 1 |  |  |  |  |  |  |
| FR13 | 2 | M | May 2019 | Białowieża | Stomach |  | 1 |  |  |  |  |  |  |  |  |  |
|  |  |  |  |  | Intestines |  | 1 |  |  |  |  |  |  |  |  |  |
| PY02 | 2 | M | January 2022 | Białystok | Stomach |  |  |  |  | 1 |  |  |  |  |  |  |
| PY04 | 2 | F | October 2019 | Białowieża | Stomach | 1 |  |  |  |  |  |  |  |  |  |  |
| PY05 | 2 | F | October2020 | Białystok | Intestines |  |  |  |  | 1 |  |  |  |  |  |  |
| PY08 | 2 | M | April 2023 | Białowieża | Stomach |  |  | 1 |  |  |  |  |  |  |  |  |
| SP01 | 2 | M | June 2022 | Białystok | Intestines |  |  | 1 |  |  |  |  |  |  |  |  |
| SP04 | 1 | F | October 2022 | Białystok | Stomach | 3 |  |  |  |  |  |  |  |  |  |  |
| SP07 | 1 | F | October 2022 | Białystok | Stomach |  |  |  |  | 1 |  |  |  |  |  |  |
|  |  |  |  |  | Intestines |  |  | 1 |  |  |  |  |  |  |  |  |
| SP08 | 1 | F | September 2022 | Białystok | Stomach |  |  |  |  | 1 |  |  |  |  |  |  |
|  |  |  |  |  | Intestines |  |  |  |  | 1 |  |  |  |  |  |  |
| SP09 | 1 | F | September 2022 | Białystok | Intestines |  |  | 2 |  |  |  |  |  |  |  |  |
| SP10 | 2 | F | February 2022 | Białystok | Stomach |  |  |  | 1 |  |  |  |  |  |  |  |
| SP11 | 2 | F | April 2019 | Białystok | Stomach | 1 |  |  |  | 1 |  |  |  |  |  |  |
|  |  |  |  |  | Intestines |  |  | 1 |  |  |  |  |  |  |  |  |
| SP12 | 1 | F | October 2019 | Suraż | Intestines |  |  |  |  |  |  | 2 |  |  |  |  |
| SP13 | 1 | F | November 2019 | Gać | Intestines |  |  |  |  | 1 |  |  |  |  |  |  |
| SP14 | 1 | F | February 2022 | Białystok | Stomach |  |  |  | 1 |  |  |  |  |  |  |  |
|  |  |  |  |  | Intestines |  |  |  | 1 |  |  |  |  |  |  |  |
| SP15 | 1 | M | 2020 | Białystok | Stomach |  |  |  | 2 | 2 |  |  | 1 |  |  |  |
| SP16 | 1 | F | 2020 | Białystok | Stomach | 1 |  |  |  |  |  |  |  |  |  |  |
|  |  |  |  |  | Intestines |  |  |  |  | 1 |  |  |  |  |  |  |
| SP18 | 2 | F | June 2022 | Białystok | Intestines |  |  |  |  | 1 |  |  |  |  |  |  |
| SP19 | 2 | F | March 2023 | Białystok | Intestines |  |  |  |  |  |  |  |  | 1 |  |  |
| SP22 | 2 | M | April 2023 | Białowieża | Stomach |  |  | 1 |  |  |  |  |  |  |  |  |
|  |  |  |  |  | Intestines |  |  | 2 |  |  |  |  |  |  |  |  |
| PM06 | 1 | M | October 2022 | Białystok | Intestines |  |  |  |  |  |  |  |  |  |  | 1 |
| CY13 | 1 | - | December 2022 | Białystok | Intestines |  |  |  |  |  |  |  |  | 1 |  |  |
| PA15 | 1 | F | October 2020 | Jurowce | Intestines |  |  |  |  |  |  | 2 |  |  |  |  |

**Table A.2. Microscopic anthropogenic litter characteristics in relation to size class found in the proventriculus and gizzard of the studied species. European Goldfinch is omitted since there was no microscopic anthropogenic litter observed in the gastrointestinal tracts of analyzed individuals. The fibers category describes the size of all fiber types observed in studied samples.**

| **Size (μm)** | **Greenfinch** | | **Hawfinch** | | **Chaffinch** | | **Siskin** | | **Bullfinch** | | **Great Tit** | | **Blue Tit** | | **Coal Tit** | |
| --- | --- | --- | --- | --- | --- | --- | --- | --- | --- | --- | --- | --- | --- | --- | --- | --- |
|  | **MP/AF** | **SiO_2_** | **MP/AF** | **SiO_2_** | **MP/AF** | **SiO_2_** | **MP/AF** | **SiO_2_** | **MP/AF** | **SiO_2_** | **MP/AF** | **SiO_2_** | **MP/AF** | **SiO_2_** | **MP/AF** | **SiO_2_** |
| 80- 100 |  |  |  |  |  |  |  |  |  |  | 1 |  | 1 |  |  |  |
| 100-199 | 1 |  |  | 5 |  |  | 1 |  |  |  |  |  |  |  | 1 |  |
| 200-299 | 1 |  | 1 | 4 |  |  | 2 |  |  | 1 |  |  |  |  |  |  |
| 300-399 |  |  |  | 1 |  |  |  |  |  |  |  |  |  |  |  |  |
| 400-499 |  |  |  | 2 |  |  | 1 | 2 | 1 |  |  |  |  |  |  |  |
| 500-599 |  |  | 1 | 1 |  |  | 2 | 1 |  |  |  |  |  |  |  |  |
| 600-699 | 1 |  | 1 | 1 |  |  | 3 | 2 |  |  |  |  |  |  |  |  |
| 700-799 | 2 |  | 2 | 2 |  |  | 1 |  | 1 |  |  |  |  |  |  |  |
| 800-899 |  | 1 | 2 |  |  |  |  |  |  |  |  |  |  |  |  |  |
| 900-999 |  |  | 2 |  |  |  | 1 |  |  |  |  |  |  |  |  |  |
| 1000-1099 |  |  |  |  |  |  |  |  |  |  |  |  |  |  |  |  |
| 1100-1199 |  |  | 2 |  |  |  | 4 |  |  |  |  |  |  |  |  |  |
| 1200-1299 |  |  | 3 | 1 |  |  |  |  |  |  |  |  |  |  |  |  |
| 1300-1399 |  |  | 1 |  |  |  |  |  |  |  |  |  |  |  |  |  |
| 1400-1499 | 1 |  |  |  |  |  | 1 |  |  |  |  |  |  |  |  |  |
| 1500-1599 |  |  |  |  |  |  | 1 |  |  |  |  |  |  |  |  |  |
| 1600-1699 |  |  |  |  | 1 |  |  |  |  |  |  |  |  |  |  |  |
| 1700-1799 |  |  |  |  |  |  | 1 |  |  |  |  |  |  |  |  |  |
| 1800-1899 |  |  | 1 |  |  |  | 1 |  |  |  |  |  |  |  |  |  |
| 1900-1999 |  |  |  |  |  |  |  |  |  |  |  |  |  |  |  |  |
| 2000-2099 |  |  |  |  |  |  |  |  |  |  |  |  |  |  |  |  |
| 2100-2199 |  |  | 2 |  |  |  | 2 |  |  |  |  |  |  |  |  |  |
| 2200-2299 |  |  |  |  |  |  |  |  |  |  |  |  |  |  |  |  |
| 2300-2399 |  |  | 1 |  |  |  |  |  |  |  |  |  |  |  |  |  |
| 2400-2499 |  |  |  |  |  |  |  |  |  |  |  |  |  |  |  |  |
| 2500-2599 |  |  |  |  |  |  |  |  |  |  |  |  |  |  |  |  |
| 2600-2699 |  |  |  |  |  |  |  |  |  |  |  |  |  |  | 1 |  |
| 2700-2799 |  |  |  |  |  |  |  |  |  |  |  |  |  |  |  |  |
| 2800-2899 |  |  |  |  |  |  |  |  |  |  |  |  |  |  |  |  |
| 2900-2999 |  |  |  |  |  |  |  |  |  |  |  |  |  |  |  |  |
| 3000-3099 |  |  |  |  |  |  |  |  |  |  |  |  |  |  |  |  |
| 3100-3199 |  |  |  |  |  |  |  |  |  |  |  |  |  |  |  |  |
| 3200-3299 |  |  | 1 |  |  |  |  |  |  |  |  |  |  |  |  |  |
| 3300-3399 | 1 |  |  |  |  |  |  |  |  |  |  |  |  |  |  |  |
| 3400-3499 |  |  |  |  |  |  |  |  |  |  |  |  |  |  |  |  |
| 3500-3599 |  |  |  |  |  |  |  |  |  |  |  |  |  |  |  |  |
| 3600-3699 |  |  |  |  |  |  |  |  |  |  |  |  |  |  |  |  |
| 3700-3799 |  |  |  |  |  |  |  |  |  |  |  |  |  |  |  |  |
| 3800-3899 | 1 |  |  |  |  |  |  |  |  |  |  |  |  |  |  |  |
| 3900-3999 |  |  |  |  |  |  |  |  |  |  |  |  |  |  |  |  |
| 4000-4099 |  |  |  |  |  |  |  |  |  |  |  |  |  |  |  |  |
| 4100-4199 |  |  |  |  |  |  |  |  | 1 |  |  |  |  |  |  |  |
| 4200-4299 |  |  |  |  |  |  |  |  |  |  |  |  |  |  |  |  |
| 4300-4399 |  |  |  |  |  |  |  |  |  |  |  |  |  |  |  |  |
| 4400-4499 |  |  |  |  |  |  |  |  |  |  |  |  |  |  |  |  |
| 4500-4599 |  |  |  |  |  |  |  |  |  |  |  |  |  |  |  |  |
| 4600-4699 |  |  |  |  |  |  |  |  |  |  |  |  |  |  |  |  |
| 4700-4799 |  |  |  |  |  |  |  |  |  |  |  |  |  |  |  |  |
| 4800-4899 |  |  |  |  |  |  |  |  |  |  |  |  |  |  |  |  |
| 4000-4999 |  |  |  |  |  |  |  |  |  |  |  |  |  |  |  |  |

**Table A.3.** Color characteristics of microscopic anthropogenic litter found in the stomach and intestines of the analyzed individuals. Litter codes: SiO_2_ – sand grain (quartz); Ce – natural cellulose; ACe – anthropogenic cellulose; Co – natural cotton; ACo – anthropogenic cotton; Ra – rayon; VI – viscose; CNT – carbon nanotubes; PET – polyethylene terephthalate; PE – polyethylene; PS – polystyrene.

| **Colour** | **SiO_2_** | **Ce** | **ACe** | **Co** | **ACo** | **Ra** | **Vi** | **CNT** | **PET** | **PE** | **PS** |
| --- | --- | --- | --- | --- | --- | --- | --- | --- | --- | --- | --- |
| Greenfinch | | | | | | | | | | | |
| Black | 1 | 0 | 7 | 0 | 0 | 0 | 0 | 0 | 0 | 0 | 0 |
| Blue | 0 | 0 | 1 | 0 | 0 | 0 | 0 | 0 | 0 | 0 | 0 |
| Green | 0 | 0 | 0 | 0 | 0 | 0 | 0 | 0 | 0 | 0 | 0 |
| Transparent | 0 | 0 | 0 | 0 | 0 | 0 | 0 | 0 | 0 | 0 | 0 |
| Hawfinch | | | | | | | | | | | |
| Black | 0 | 0 | 2 | 0 | 10 | 2 | 0 | 0 | 0 | 0 | 0 |
| Blue | 0 | 0 | 0 | 0 | 1 | 0 | 0 | 0 | 1 | 0 | 0 |
| Green | 0 | 0 | 0 | 0 | 0 | 0 | 0 | 0 | 0 | 0 | 0 |
| Transparent | 17 | 0 | 0 | 3 | 1 | 0 | 0 | 0 | 0 | 3 | 0 |
| Chaffinch | | | | | | | | | | | |
| Black | 0 | 0 | 0 | 0 | 0 | 0 | 0 | 0 | 0 | 0 | 0 |
| Blue | 0 | 0 | 0 | 0 | 1 | 0 | 0 | 0 | 0 | 0 | 0 |
| Green | 0 | 0 | 0 | 0 | 0 | 0 | 0 | 0 | 0 | 0 | 0 |
| Transparent | 0 | 4 | 0 | 0 | 0 | 0 | 0 | 0 | 0 | 0 | 0 |
| Siskin | | | | | | | | | | | |
| Black | 0 | 0 | 3 | 0 | 6 | 0 | 2 | 1 | 0 | 0 | 0 |
| Blue | 0 | 0 | 4 | 0 | 2 | 0 | 0 | 0 | 1 | 0 | 0 |
| Green | 0 | 0 | 0 | 0 | 0 | 0 | 0 | 0 | 0 | 0 | 0 |
| Transparent | 5 | 0 | 1 | 5 | 1 | 0 | 0 | 0 | 0 | 0 | 0 |
| Bullfinch | | | | | | | | | | | |
| Black | 0 | 0 | 1 | 0 | 2 | 0 | 0 | 0 | 0 | 0 | 0 |
| Blue | 0 | 0 | 0 | 0 | 0 | 0 | 0 | 0 | 0 | 0 | 0 |
| Green | 0 | 0 | 0 | 0 | 0 | 0 | 0 | 0 | 0 | 0 | 0 |
| Transparent | 1 | 0 | 0 | 0 | 0 | 0 | 0 | 0 | 0 | 0 | 0 |
| Great Tit | | | | | | | | | | | |
| Black | 0 | 0 | 0 | 0 | 0 | 0 | 0 | 0 | 0 | 0 | 0 |
| Blue | 0 | 0 | 0 | 0 | 0 | 0 | 0 | 0 | 0 | 0 | 1 |
| Green | 0 | 0 | 0 | 0 | 0 | 0 | 0 | 0 | 0 | 0 | 0 |
| Transparent | 0 | 0 | 0 | 0 | 0 | 0 | 0 | 0 | 0 | 0 | 0 |
| Blue Tit | | | | | | | | | | | |
| Black | 0 | 0 | 0 | 0 | 0 | 0 | 0 | 0 | 0 | 0 | 0 |
| Blue | 0 | 0 | 0 | 0 | 0 | 0 | 0 | 0 | 0 | 0 | 0 |
| Green | 0 | 0 | 0 | 0 | 0 | 0 | 0 | 0 | 1 | 0 | 0 |
| Transparent | 0 | 0 | 0 | 0 | 0 | 0 | 0 | 0 | 0 | 0 | 0 |
| Coal Tit | | | | | | | | | | | |
| Black | 0 | 0 | 0 | 0 | 0 | 0 | 1 | 0 | 0 | 0 | 0 |
| Blue | 0 | 0 | 0 | 0 | 0 | 0 | 0 | 0 | 0 | 0 | 0 |
| Green | 0 | 0 | 0 | 0 | 0 | 0 | 1 | 0 | 0 | 0 | 0 |
| Transparent | 0 | 0 | 0 | 0 | 0 | 0 | 0 | 0 | 0 | 0 | 0 |
